# Expression of hypoxia-inducible factor 1 alpha and its downstream targets in fibroepithelial tumors of the breast

**DOI:** 10.1186/bcr1296

**Published:** 2005-08-05

**Authors:** Arno Kuijper, Petra van der Groep, Elsken van der Wall, Paul J van Diest

**Affiliations:** 1 Department of Pathology, VU University Medical Center, Amsterdam, The Netherlands; 2 Department of Pathology, University Medical Center Utrecht, The Netherlands; 3 Division of Internal Medicine and Dermatology, University Medical Center Utrecht, The Netherlands

## Abstract

**Introduction:**

Hypoxia-inducible factor 1 (HIF-1) alpha and its downstream targets carbonic anhydrase IX (CAIX) and vascular endothelial growth factor (VEGF) are key factors in the survival of proliferating tumor cells in a hypoxic microenvironment. We studied the expression and prognostic relevance of HIF-1α and its downstream targets in phyllodes tumors and fibroadenomas of the breast.

**Methods:**

The expression of HIF-1α, CAIX, VEGF and p53 was investigated by immunohistochemistry in a group of 37 primary phyllodes tumors and 30 fibroadenomas with known clinical follow-up. The tumor microvasculature was visualized by immunohistochemistry for CD31. Proliferation was assessed by Ki67 immunostaining and mitotic counts. Being biphasic tumors, immunoquantification was performed in the stroma and epithelium.

**Results:**

Only two fibroadenomas displayed low-level stromal HIF-1α reactivity in the absence of CAIX expression. Stromal HIF-1α expression was positively correlated with phyllodes tumor grade (*P *= 0.001), with proliferation as measured by Ki67 expression (*P *< 0.001) and number of mitoses (*P *< 0.001), with p53 accumulation (*P *= 0.003), and with global (*P *= 0.015) and hot-spot (*P *= 0.031) microvessel counts, but not with CAIX expression. Interestingly, concerted CAIX and HIF-1α expression was frequently found in morphologically normal epithelium of phyllodes tumors. The distance from the epithelium to the nearest microvessels was higher in phyllodes tumors as compared with in fibroadenomas. Microvessel counts as such did not differ between fibroadenomas and phyllodes tumors, however. High expression of VEGF was regularly found in both tumors, with only a positive relation between stromal VEGF and grade in phyllodes tumors (*P *= 0.016). Stromal HIF-1α overexpression in phyllodes tumors was predictive of disease-free survival (*P *= 0.032).

**Conclusion:**

These results indicate that HIF-1α expression is associated with diminished disease-free survival and may play an important role in stromal progression of breast phyllodes tumors. In view of the absence of stromal CAIX expression in phyllodes tumors, stromal upregulation of HIF-1α most probably arises from hypoxia-independent pathways, with p53 inactivation as one possible cause. In contrast, coexpression of HIF-1α and CAIX in the epithelium in phyllodes tumors points to epithelial hypoxia, most probably caused by relatively distant blood vessels. On the other hand, HIF-1α and CAIX seem to be of minor relevance in breast fibroadenomas.

## Introduction

After reaching a critical volume of several cubic millimeters, a growing tumor becomes increasingly depleted of oxygen and nutrients, and needs to adapt to its changing microenvironment. In order to survive, tumor cells must develop a vascular system and adapt their metabolism. A key regulator in this process is the transcription factor hypoxia-inducible factor 1 (HIF-1) [1], which controls the expression of several target genes. The protein product of *HIF-1 *is a heterodimer and consists of two subunits, HIF-1α and HIF-1β. Under normoxic conditions the HIF-1α protein is rapidly degraded. O_2_-dependent hydroxylation of proline residues in HIF-1α causes binding of the von Hippel–Lindau tumor suppressor protein, which leads to ubiquitilation and subsequent degradation by the proteasome [2,3]. Hypoxia inhibits this process, resulting in upregulation of HIF-1α and its downstream target genes [4]. On the other hand, hypoxia-independent upregulation of HIF-1α may be accomplished by loss of tumor suppressor genes such as the phosphate and tensin homolog deleted on chromosome 10 (*PTEN*) [5] and the von Hippel–Lindau tumor suppressor gene *VHL *[6], by activation of oncogenes like *v-Src *[7] and by stimulation by growth factors such as insulin-like growth factors [8,9] and epidermal growth factor [10].

It has recently been demonstrated that HIF-1α expression is of prognostic value in several types of cancer, including breast cancer [11]. A well-known target of HIF-1α is the vascular endothelial growth factor (*VEGF*) gene [12]. VEGF is a potent endothelial cell-specific mitogen and is a major participant in the process of angiogenesis, resulting in the formation of microvessels. Both VEGF expression [13,14] and microvessel density [15,16] are established prognosticators in many types of cancer. Upregulation of carbonic anhydrase 9 (*CA9*) gene expression was found to be dependent on HIF-1α [17]. The protein product of the *CA9 *gene, carbonic anhydrase IX (CAIX), catalyzes the hydration of carbon dioxide to carbonic acid and contributes to acidification of the surrounding microenvironment. CAIX is constitutively expressed in cells lining the alimentary tract [18]. Because of its correlation with lowered pO_2 _in carcinoma of the cervix, CAIX expression is regarded as an intrinsic marker of hypoxia [19]. Furthermore, CAIX expression has recently been related to poor outcome in breast cancer [20]. It therefore seems that HIF-1α and its downstream targets play pivotal roles in the development and progression of cancer.

Breast fibroadenoma and phyllodes tumor are both fibroepithelial tumors; that is, they are composed of an epithelial component and a stromal component. The distinction between both tumors may be difficult [21]. The behavior of fibroadenomas is benign, however, in contrast to phyllodes tumors that can recur and can even metastasize. In a small number of cases, fibroadenoma may progress to phyllodes tumor [22,23]. Little is known of the mechanisms by which these tumors maintain a steady supply of nutrients as they grow. Few studies have addressed expression patterns of angiogenic growth factors in fibroepithelial breast tumors. Expression of basic fibroblast growth factor (basic FGF), FGF receptor and VEGF was found at higher levels in stroma of phyllodes tumors in comparison with fibroadenomas [24]. Unfortunately, no information on the epithelial component was provided in this work. Expression patterns of platelet-derived growth factor (PDGF) and the PDGF receptor suggest the presence of autocrine loops in stroma and paracrine stimulation of stroma by the epithelium [25]. A similar autocrine and paracrine loop has been described for acidic FGF and FGF receptor 4 in fibroadenomas [26]. These studies suggest that stromal proliferation may be stimulated by secretion of mitogens by the epithelial compartment. Interestingly, hypoxia may stimulate both FGF and PDGF expression [27,28]. It would therefore be interesting to evaluate the role of HIF-1α and its downstream effectors in the tumorigenesis of biphasic breast tumors.

We therefore studied expression of HIF-1α and its downstream targets VEGF and CAIX in breast phyllodes tumors of various grades and in fibroadenomas. Furthermore, since HIF-1α seems to play a major role in the process of angiogenesis, we evaluated the microvascular network by counting of CD31-positive microvessels. Proliferation, as an important functional end point of various carcinogenic processes, was assessed by mitotic counts and Ki67 expression. Since HIF-1α degradation may be promoted by wild-type p53 [29], we evaluated the relation between p53 and HIF-1α expression in phyllodes tumors. Finally, the prognostic value of HIF-1α, its downstream effectors and microvessel counts was also assessed for phyllodes tumors.

## Materials and methods

### Tissue samples

Formaldehyde-fixed and paraffin-embedded tissue samples were retrieved from the archives of our hospitals. A total of 37 primary phyllodes tumors and 30 fibroadenomas were acquired. The presence of epithelial proliferative changes was noted. Phyllodes tumors were graded as benign, borderline or malignant based on the degree of stromal cellularity, the degree of stromal overgrowth, the degree of cellular atypia, invasiveness of the tumor margin and the mitotic activity index as described previously in detail by Moffat and colleagues [30]. Mitotic figures were counted using established criteria in 10 consecutive high-power fields at a 400 × magnification [31]. As the grading of phyllodes tumors may be complicated by intratumoral heterogeneity, tumors were graded in the most unfavorable areas provided they comprised at least 10% of the total tumor area [30]. Clinical data were gathered by studying medical records.

### Immunohistochemistry

Four-micrometer sections were cut and mounted on coated slides. After deparrafinization and rehydration, sections were immersed in methanol containing 0.3% hydrogen peroxide to stop endogenous peroxidase activity. No antigen retrieval was necessary for CAIX staining.

Antigen retrieval for HIF-1α was performed in target retrieval solution (DAKO, Glostrup, Denmark) in a water bath at 97°C for 45 min, and the remaining antigens were unmasked by microwaving the slides for 10–15 min in citrate buffer (pH 6.0). The following panel of mouse monoclonal antibodies was used: VEGF (1:50, R&D Systems, Abingdon, U.K), HIF-1α (1:500; BDTransduction Laboratories, Lexington, Kentucky, USA), CAIX (M75, 1:50; kind gift from Dr S Pastorekova (Institute of Virology, Academy of Science, Bratislava, Slovakia)), CD31 (JC/70 A, 1:40; DAKO), Ki67 (MIB-1, 1:40; DAKO) and p53 (DO-7, 1:500; DAKO). VEGF, Ki67 and p53 were incubated overnight at 4°C, the incubation time for HIF-1α and CAIX was 30 min at room temperature, and CD31 was incubated for 1 hour at room temperature. VEGF, Ki67 and p53 were detected by application of a secondary biotinylated rabbit anti-mouse antibody (diluted 1:500; DAKO) followed by incubation with avidin–biotin-peroxidase complex (1:200 dilution; DAKO). The Catalyzed Signal Amplification system (DAKO) was used to detect HIF-1α, CD31 was visualized with the Ultravision system (Labvision) and CAIX was detected with the Envision system peroxidase (DAKO). All stainings were developed with 3,3'-diaminobenzidine tetrahydrochloride. Counter staining was performed with hematoxylin. Negative controls were obtained by omitting the primary antibody, and appropriate positive controls were included throughout.

As fibroadenomas and phyllodes tumors are biphasic tumors, immunostainings were scored in both stroma and the epithelium. Immunoquantification was performed simultaneously by two observers (AK and PJvD) behind a double-headed microscope. The percentage of positive staining nuclei for HIF-1α, Ki67 and p53 was estimated on a continuous scale, regarding only homogeneously and darkly stained nuclei as positive. CAIX expression was scored as positive or negative, scoring a case positive when membranous staining in any amount was present. Cytoplasmic VEGF staining was scored semi-quantitatively in four categories from 0 to +++, with category 0 expressing no positive staining, category + showing focal or diffuse weak staining, and categories ++ and +++ displaying focal or widespread strong staining, respectively. Counting of CD31-positive microvessels was performed in the microvessel hot-spot in four adjacent fields of vision at 400 × magnification as described elsewhere [15,16]. In addition, a global microvessel density was acquired by counting of vessels in 10 diagonally adjacent fields at a magnification of 400 × from a random starting point generated semi-automatically by the QPRODIT interactive digitizing video overlay system (Leica, Cambridge, UK).

For statistical analysis, stainings for Ki67 and p53 were divided into high and low using 10% positive staining as the cut-off [32]. Categories 0 and + staining for VEGF were grouped as low expression and ++/+++ was stated high, and microvessel counts were dichotomized using the median value. As determined by staining of normal breast tissue, preinvasive breast lesions and invasive breast cancer, HIF-1α overexpression was defined as ≥1% positive staining nuclei [33].

The mean shortest distances from microvessels to the epithelial basal membrane were determined with the QPRODIT system. Using CD31-stained sections, the distances of the epithelium to the nearest microvessel were measured between manually placed markers. Means were calculated from a minimum of 50 measurements per case with fields of view selected according to a systematic random sampling method [34].

### Statistical analysis

All statistical analyses were performed with SPSS software (SPSS, Chicago, IL, USA). Differences in expression of HIF-1α, of Ki67, of VEGF and of CAIX and the microvessel density between fibroadenomas and different grades of phyllodes tumors were investigated by the chi-square test. Correlations between markers were evaluated using Fisher's exact test. The clinical endpoint for survival analysis of phyllodes tumors was local or distant recurrence (disease-free survival). Wide local excision is the preferred treatment for phyllodes tumors. Patients treated by primary mastectomy were therefore excluded from survival analysis since these would bias results due to strongly reduced chances of recurrence. Kaplan–Meier curves were plotted and differences between the curves were evaluated with the log-rank test. *P *values below 0.05 were regarded as significant.

## Results

### Patient characteristics

Thirty fibroadenomas were analyzed. The mean age of patients was 33.7 ± 10.5 years and the mean tumor size was 1.6 ± 0.6 cm. Ten fibroadenomas (33%) harbored epithelial proliferative changes.

A total of 18 benign primary phyllodes tumors, eight borderline primary phyllodes tumors and 11 malignant primary phyllodes tumors were identified. Epithelial hyperplasia of the ductal type, mostly focal, was found in 16 cases (43%) and was not related to grade. The mean age of patients with benign, borderline and malignant phyllodes tumors was 44.4 ± 17.4, 57.9 ± 12.8 and 54.3 ± 12.9 years, respectively (*P *= 0.073). Mean tumor sizes were 4.8 ± 2.4, 7.1 ± 7.0 and 4.8 ± 2.5 cm for benign, borderline and malignant phyllodes tumors, respectively (*P *= 0.924). Patients with phyllodes tumors were older and had larger tumors (*P *< 0.001 both) compared with those patients with fibroadenomas. Five patients with phyllodes tumors were treated by primary mastectomy, the remainder by local excision. Excision was incomplete for 13 phyllodes tumors, whereas for six phyllodes tumors this information could not be retrieved.

### Differences between fibroadenomas and phyllodes tumors

**Figure 1:**
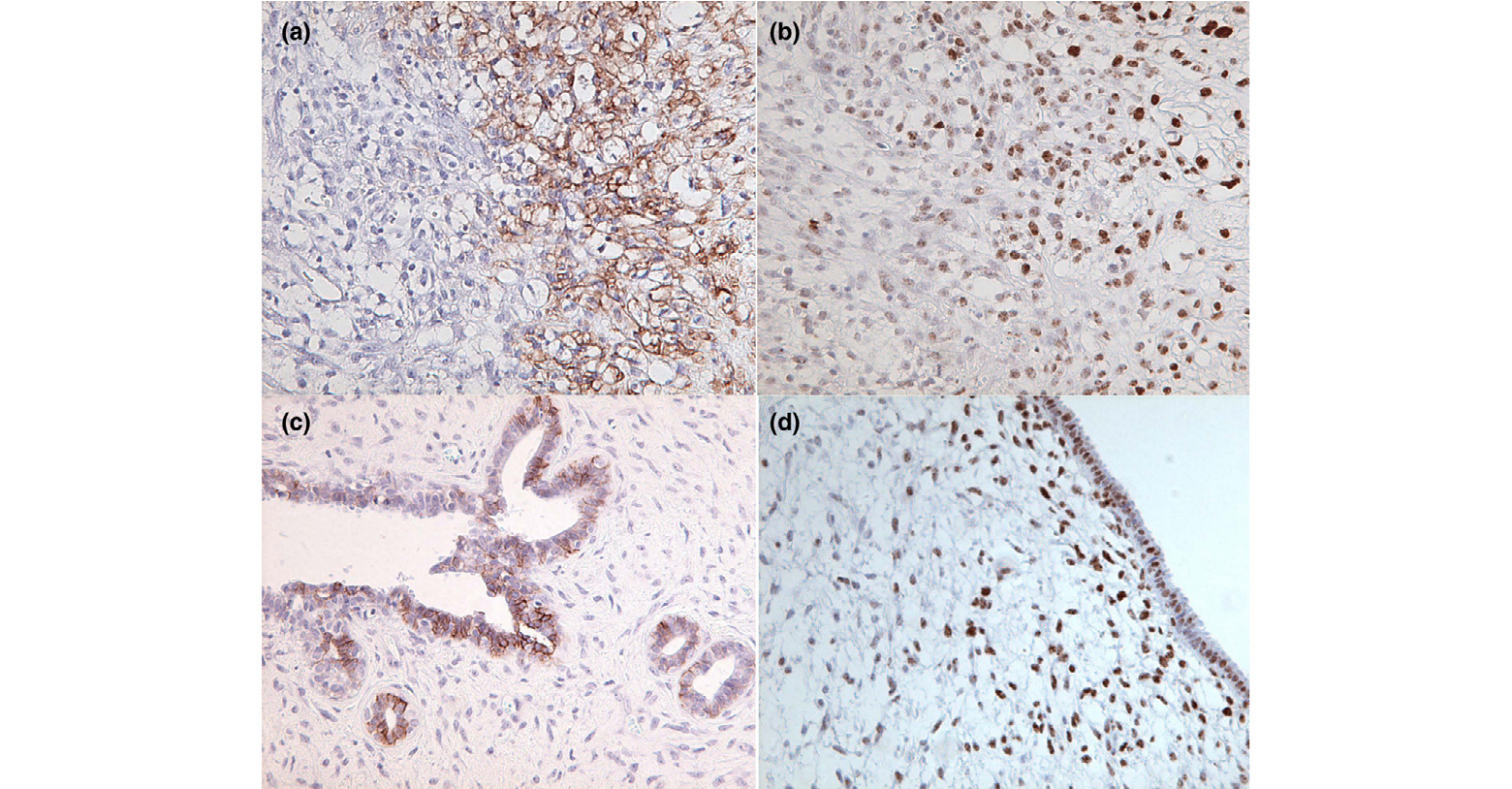
Examples of immunostaining for hypoxia-inducible factor 1 alpha (HIF-1α) and carbonic anhydrase IX (CAIX) in breast phyllodes tumors. **(a) **Malignant phyllodes tumor with stromal CAIX expression. **(b) **Same tumor as (a) with topographically overlapping HIF-1α overexpression. **(c) **Benign phyllodes tumor with CAIX-positive staining epithelium. **(d) **Borderline phyllodes tumor with HIF-1α overexpression in normal appearing epithelium and in subepithelial stroma.

Table 1 summarizes the differences in immunostaining between fibroadenomas and phyllodes tumors. Stromal and epithelial overexpression of HIF-1α were found almost exclusively in phyllodes tumors (*P *= 0.001 and *P *< 0.001, respectively; Fig. 1b,d). Only two fibroadenomas with low-level stromal HIF-1α immunoreactivity (1% and 2% positive nuclei) were found without concerted CAIX expression. Stromal and epithelial CAIX expression was only seen in phyllodes tumors (Fig. 1a,c). High expression of VEGF was regularly found in both fibroadenomas and phyllodes tumors. As a result, no differences in stromal and epithelial VEGF expression were found between both tumors.

**Figure 2:**
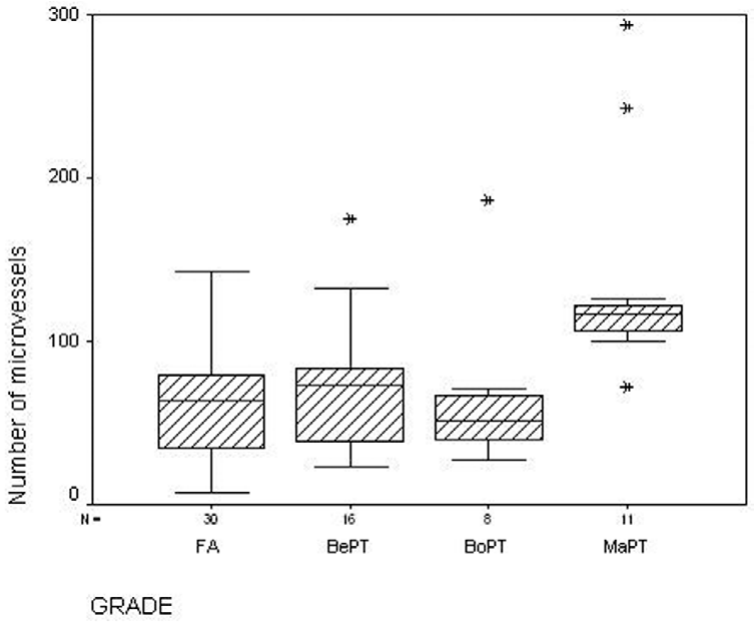
Hot-spot microvessel counts in fibroadenomas and phyllodes tumors. Boxplot showing the large overlap in the numbers of microvessels between fibroadenomas (FA), benign phyllodes tumors (BePT) and borderline phyllodes tumors (BoPT) when counted in the hot-spot. MaPT, malignant phyllodes tumor.

Many small microvessels were concentrated around the epithelium of fibroadenomas. In phyllodes tumors, microvessels lacked such a peri-epithelial preference and were distributed more evenly throughout the tumor. The number of microvessels as such, counted by both methods, did not differ significantly between fibroadenomas and phyllodes tumors. Indeed, when regarding microvessel counts on a continuous scale, a large overlap was observed between fibroadenomas, benign phyllodes tumors and borderline phyllodes tumors (Fig. 2). In five fibroadenomas and 10 phyllodes tumors, of which five had stretches of HIF-1α-positive epithelium, the mean shortest distance between microvessels and the epithelial basal membrane was 50 ± 11 μm for fibroadenomas and was 83.3 ± 16 μm for phyllodes tumors (*P *= 0.005). Because of the focal staining patterns resulting in methodological problems and low numbers, phyllodes tumors with HIF-1α-negative and HIF-1α-positive epithelium were not separately analyzed here. Due to these methodological problems the results should be interpreted with caution.

Fibroadenomas and phyllodes tumors did not differ with regard to the presence of epithelial proliferative changes (*P *= 0.458).

### Differences between phyllodes tumor grades

Correlations between the grade of phyllodes tumor and HIF-1α and its downstream targets, microvessels and proliferation are summarized in Table 2. Stromal HIF-1α overexpression in phyllodes tumors was strongly correlated with tumor grade (*P *= 0.001), with all malignant tumors displaying overexpression. Necrosis could be detected in only one malignant phyllodes tumor, with no typical peri-necrotic HIF-1α overexpression pattern. Epithelial HIF-1α overexpression was not related to grade (*P *= 0.323). Only two malignant phyllodes tumors displayed stromal CAIX expression (*P *= 0.090). Epithelial CAIX expression was seen more often than stromal CAIX expression, but was not correlated to grade (*P *= 0.735). Overexpression of HIF-1α and expression of CAIX in the epithelial component were both not related to hyperplasia and were mostly found in normal appearing, two-layered epithelium.

A statistically significant difference in the number of microvessels was found between grades, both when counted in the hot-spot and by the global method (*P *= 0.003 and *P *= 0.002, respectively). The number of microvessels was strongly increased in malignant tumors, as compared with benign and borderline tumors (Fig. 2). High VEGF expression in the stromal component displayed a positive relation with tumor grade (*P *= 0.016).

HIF-1α expression in both compartments of phyllodes tumors was not significantly related to tumor size, both with size cut-off at the median and when used as a continuous variable (data not shown).

### Coexpression of markers

The relations between HIF-1α expression and its downstream targets, microvessels and proliferation markers in the stromal component of fibroepithelial tumors are presented in Table 3. Since only two cases showed stromal CAIX expression, the relation between stromal CAIX and stromal HIF-1α overexpression did not reach statistical significance (*P *= 0.098). The relation between HIF-1α overexpression and strong VEGF expression in stroma reached borderline significance (*P *= 0.098). Global (*P *= 0.015) and hot-spot (*P *= 0.031) microvessel counts were both related to stromal HIF-1α overexpression. Overexpression of HIF-1α in stroma was correlated with proliferation as measured by high stromal Ki67 expression (*P *< 0.001) and by high mitotic activity index (*P *< 0.001). Stromal overexpression of HIF-1α and p53 were also strongly associated (*P *= 0.003).

Epithelial HIF-1α overexpression was related to epithelial CAIX expression (*P *= 0.014). There was no relation between HIF-1α overexpression in the epithelial component and VEGF or Ki67 expression. Epithelial HIF-1α overexpression was related to increased stromal proliferation (Ki67, *P *= 0.006; mitotic activity index, *P *= 0.004).

### Survival analysis

**Figure 3:**
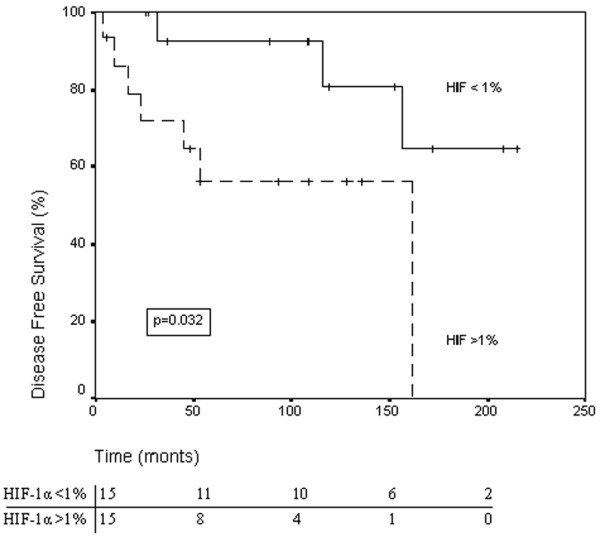
Disease-free survival according to hypoxia-inducible factor 1 alpha (HIF-1α) status. The Kaplan–Meier survival curve illustrating disease-free survival for patients with breast phyllodes tumors with high expression (≥1%) versus low expression (<1%) of HIF-1α in the stromal component. Numbers of patients at risk at different time points are displayed below the horizontal axis.

As already mentioned, patients treated by mastectomy were excluded from survival analysis. Two patients were lost to follow-up. Ten of the remaining 30 tumors recurred. Most were local recurrences, but one malignant tumor metastasized to the lung. By univariate analysis of survival we found an inverse correlation between stromal HIF-1α overexpression (*P *= 0.032; Fig. 3), tumor grade (*P *= 0.039), stromal Ki67 overexpression (*P *= 0.028) and stromal p53 overexpression (*P *< 0.001) and disease-free survival, as presented in Table 4. HIF-1α expression did not add to the prognostic power of p53 expression alone. The tumor size (median, 3 cm; *P *= 0.88), age (*P *= 0.36), the mitotic activity index (*P *= 0.09), the microvessel counts (hot-spot *P *= 0.54; global *P *= 0.25), the margin status (*P *= 0.23), the VEGF expression (stromal *P *= 0.96; epithelial *P *= 0.11), the CAIX expression (stromal *P *= 0.46; epithelial *P *= 0.16) and the epithelial HIF-1α expression (*P *= 0.76) were not of prognostic relevance. Surprisingly, epithelial overexpression of Ki67 also had prognostic power (*P *= 0.011). This is of no relevance, however, since only two cases showed epithelial overexpression of Ki67.

## Discussion

Evidence is mounting that HIF-1α and its downstream targets play pivotal roles in the development and progression of many types of human cancers. This is the first study evaluating HIF-1α and CAIX expression in breast phyllodes tumors. We found that stromal HIF-1α overexpression predicts the prognosis of, and may play an important role in, the stromal progression of phyllodes tumors. Surprisingly, we regularly found concerted HIF-1α and CAIX expression in normal appearing epithelium of phyllodes tumors. Since only two fibroadenomas displayed low-level stromal HIF-1α immunoreactivity and none expressed CAIX, HIF-1α and CAIX seem to be of little relevance in these tumors.

HIF-1α overexpression is induced by hypoxia and by oxygen-independent mechanisms. In our study, stromal HIF-1α overexpression was related to the grade of phyllodes tumors, with a marked increase from borderline to malignant grade. Benign and borderline tumors showed comparable levels of HIF-1α overexpression. We only found two fibroadenomas with low-level expression of HIF-1α in the stroma, in agreement with Zhong and colleagues, who found all their fibroadenomas to be negative [35]. By clonality analysis we previously demonstrated that fibroadenomas may progress to phyllodes tumors by clonal expansion of stroma [22]. Perhaps positive stromal HIF-1α staining in a fibroadenoma may reflect an increased intrinsic capacity to progress to phyllodes tumor. The fibroadenomas with stromal HIF-1α positivity were microscopically inconspicuous, however.

In several types of cancer, hypoxia-induced HIF-1α expression seems to be characterized by a peri-necrotic distribution, whereas oxygen-independent overexpression results in a diffuse pattern of HIF-1α immunoreactivity [11,36,37]. Necrosis was found in the stromal component of only one malignant phyllodes tumor. Furthermore, stromal HIF-1α overexpression was not accompanied by an increase in CAIX expression, which is regarded as a marker of hypoxia [19]. Indeed, our group recently showed that, in contrast to homogeneous/diffuse HIF-1α expression, necrosis-related/focal HIF-1α expression is accompanied by CAIX expression [38]. All this suggests that HIF-1α upregulation in stroma of phyllodes tumors is normoxic and may be caused by changes in the stromal expression of oncogenes, tumor suppressor genes or growth factors. Wild-type p53 has been shown to promote MDM2-mediated ubiquitination of HIF-1α [29]. Therefore, p53 inactivation by gene mutation has been implicated in increased HIF-1α expression. We showed that aberrant expression of cell-cycle protein p53 only occurred in the stromal component. The transition from borderline to malignant phyllodes tumors was accompanied by a strong increase in stromal p53 expression, similar to stromal HIF-1α expression. Furthermore, we found a significant relation between stromal p53 and HIF-1α overexpression.

Another candidate for hypoxia-independent upregulation of HIF-1α in phyllodes tumors is PDGF. Overexpression of PDGF was found in 24% of phyllodes tumors, but not in fibroadenomas [25]. Interestingly, PDGF may induce HIF-1α expression [39]. Stromal HIF-1α and p53 expression in phyllodes tumors were predictive of disease-free survival, underlining the importance of the p53-HIF-1α axis in it's progression and clinical behavior.

Epithelial HIF-1α overexpression in phyllodes tumors was associated with CAIX expression. This suggests a causative role for epithelial hypoxia. Fibroadenomas were negative for CAIX, confirming the results of Bartosova and colleagues [40]. The mean distance from microvessels to the epithelium was significantly higher for phyllodes tumors as compared with fibroadenomas, suggesting that the hypoxic microenvironment in the epithelial component of phyllodes tumors is caused by relatively distant microvessels. Most vessels in phyllodes tumors were within 150 μm of the epithelial basal membrane, however, which is a critical distance for necrosis [41]. Still, it is conceivable that rapidly growing stroma stretches the two-layered epithelium in such way that it's oxygen supply does not keep up, resulting in a state of mild hypoxia. Indeed, a previous report detected CAIX expression 80 μm from the nearest blood vessel [42], which is comparable with the distance we found for phyllodes tumors. It is possible that the decreasing the oxygen gradient between 80 and 150 μm from the nearest vessel to the epithelium is sufficient to induce HIF-1α and CAIX expression.

The scattered foci of positive staining for HIF-1α and CAIX suggest the presence of focal mild hypoxia in the epithelial component. Microenvironmental disturbance of normal tissue by adjacent malignant disease has been described previously for HIF-1α and CAIX [40,43,44]. On the other hand, several other possible causes exist for epithelial HIF-1α overexpression. Epithelial PDGF expression, which is found in most phyllodes tumors [25], may cause HIF-1α overexpression [39]. Shpitz and colleagues found expression of HER-2/*neu *in the epithelium of 61% of phyllodes tumors [45]. It was recently demonstrated that enhanced HER-2/*neu *signaling induces HIF-1α protein expression [46]. Numerous factors interact with HIF-1 and other as yet unidentified changes in the epithelium of phyllodes tumors may cause HIF-1α overexpression.

The tumor biological significance of increased epithelial HIF-1α and CAIX expression is unclear. Morphologically, the epithelial component was mostly two-layered epithelium without atypia. Furthermore, clonality studies mostly found the epithelial component of phyllodes tumors to be polyclonal [22,23]. Moreover, phyllodes tumor metastases are composed of stroma, with only one case described in which the epithelial component also disseminated, leading to a biphasic metastasis [47]. Finally, epithelial HIF-1α and CAIX expression are not predictive of prognosis. It seems therefore that HIF-1α and CAIX expression in the epithelium of phyllodes tumors merely reflects a physiological adaptation to microenvironmental disturbance by rapidly proliferating stroma with lagging peri-epithelial angiogenesis. However, possible upregulation of growth factors in the epithelium by HIF-1α may exert an additional stimulatory force on the stromal component. Indeed, it has been suggested that in the early stages of phyllodes tumor development the epithelium secretes mitogens stimulating the stromal component [48,49]. The presence of such autocrine and paracrine loops has been described for PDGF/PDGF receptor [25]. Furthermore, stimulation of the stromal component by the epithelium was suggested previously by studying endothelin-1 expression in epithelium of phyllodes tumors [50]. Interestingly, endothelin-1 turned out to be a target of HIF-1α [51].

An abrupt increase in number of microvessels was observed in malignant phyllodes tumors, coinciding with a strong increase in stromal overexpression of HIF-1α from borderline grade to malignant grade. In contrast with a previous report [52], we found no difference in microvessel counts between benign phyllodes tumors and borderline phyllodes tumors. In addition, when regarding hot-spot and global counts we found that there was no difference between fibroadenomas, benign phyllodes tumors and borderline phyllodes tumors. This is in contrast with a previous report claiming a difference between fibroadenomas and phyllodes tumors [24]. On the other hand, Weind and colleagues found a large overlap in microvessel counts between fibroadenomas and invasive breast cancers, demonstrating that fibroadenomas are capable of producing large numbers of microvessels [53]. We found large numbers of small peri-epithelial microvessels in fibroadenomas responsible for its high numbers of microvessels. It seems that microvessel counts are not helpful in the differentiation between fibroadenomas and benign phyllodes tumors, which poses the biggest diagnostic problem.

VEGF expression in fibroadenomas did not differ from that in phyllodes tumors. Several growth factors such as FGF-4 [54], PDGF [55] and transforming growth factor beta [56] may stimulate VEGF expression. Expression of FGF [26], PDGF [25] and transforming growth factor beta [57] have been described in fibroadenomas and may contribute to HIF-1α-independent expression of VEGF. In phyllodes tumors, the relation between VEGF expression and HIF-1α reached borderline significance. Although HIF-1α most probably contributes to VEGF expression in phyllodes tumors, VEGF expression seems, at least in part, to be independent from HIF-1α.

In view of its annual incidence of 2.1 cases per 1 million women [58], we feel that our group of phyllodes tumors is reasonably sized and well composed with all grades present. Still, future investigations confirming our results in larger series are warranted. Furthermore, experiments covering a variety of molecular elements such as DNA microarray techniques may unravel the complex mechanisms underlying the presumed nonhypoxic upregulation of HIF-1α in the stromal component of phyllodes tumors and its subsequent influence on tumor progression.

## Conclusion

This is the first report on HIF-1α and CAIX expression in breast phyllodes tumors. Our results show that HIF-1α is related to diminished disease-free survival and may play an important role in stromal progression of phyllodes tumors. The significant relation between tumor grade and stromal HIF-1α overexpression underlines its importance, with all malignant tumors showing HIF-1α overexpression. Stromal HIF-1α overexpression in phyllodes tumors most probably arises from hypoxia-independent pathways, with p53 inactivation as one possible cause. Surprisingly, the normal-appearing epithelium in phyllodes tumors frequently displayed HIF-1α and CAIX expression. The distance from the nearest vessels to the epithelium was higher in phyllodes tumors as compared with fibroadenomas and a hypoxic effect seems plausible here, although the effect is of doubtful biological significance. In contrast to phyllodes tumors, HIF-1α seems of minor relevance in the tumorigenesis of fibroadenomas. Considering it's possible role in progression of the stromal component of phyllodes tumors and the fact that metastases are composed of stroma, novel therapies targeting HIF-1α [59] may contribute to the treatment of disseminated phyllodes tumor, which is poorly responsive to conventional chemotherapy and radiotherapy.

## Abbreviations

CAIX = carbonic anhydrase IX; FGF = fibroblast growth factor; HIF-1 = hypoxia-inducible factor 1; PDGF = platelet-derived growth factor; VEGF = vascular endothelial growth factor.

## Competing interests

The authors declare that they have no competing interests.

## Authors' contributions

AK was responsible for generating the hypothesis, for collecting patient material, for immunostaining, for classification of immunostainings, for statistics and for writing the manuscript. PvdG was responsible for immunostaining and for correcting the manuscript. EvdW was responsible for generating the hypothesis and for correcting the manuscript. PJvD was responsible for generating the hypothesis, for collecting patient material, for classification of immunostainings and for correcting the manuscript.

## Figures and Tables

**Table 1:** Differences in the expression of hypoxia related proteins, microvasculature and proliferation in fibroepithelial breast tumors

Marker	Fibroadenoma (*n *= 30)	Phyllodes tumor (*n *= 37)	*P *value^a^
Stroma			
HIF-1α	2 (7%)	19 (51%)	0.001
CAIX	0 (0%)	2/36 (6%)	0.497
VEGF	7 (23%)	17 (47%)	0.074
Ki67	1 (3%)	17 (47%)	<0.001
Hot-spot microvessel count	13 (43%)	22/35 (63%)	0.140
Global microvessel count	13 (43%)	20/35 (57%)	0.324
p53	0(0%)	7/36(19%)	0.014
Epithelium			
HIF-1α	0 (0%)	15/35 (43%)	<0.001
CAIX	0 (0%)	9/35 (26%)	0.003
VEGF	22 (73%)	22/36 (61%)	0.432
Ki67	2 (7%)	2/36 (6%)	1.000
p53	0(0%)	0(0%)	-

Due to empty blocks, repeated unsuccessful staining attempts or too little epithelium in a malignant tumor, numbers varied slightly for some stainings. Dichotomized values were used. ^a^Using the chi-square test, *P *values below 0.05 were regarded as significant. HIF-1α = hypoxia-inducible factor 1 alpha, CAIX = carbonic anhydrase IX, VEGF = vascular endothelial growth factor.

**Table 2:** Differences in expression of hypoxia-related proteins, microvasculature and proliferation between different grades of phyllodes tumors

Marker	Phyllodes tumor			*P *value^a^
		
	Benign (*n *= 18)	Borderline (*n *= 8)	Malignant (*n *= 11)	
Stroma				
HIF-1α	5 (31%)	3 (38%)	11 (100%)	0.001
CAIX	0 (0%)	0 (0%)	2 (18%)	0.090
VEGF	5 (31%)	3 (38%)	9 (82%)	0.016
Ki67	1 (6%)	5 (63%)	11 (100%)	<0.001
Hot-spot microvessel count	9/16 (56%)	2 (25%)	11 (100%)	0.003
Global microvessel count	8/16 (50%)	2 (25%)	11 (100%)	0.002
p53	1/18(6%)	1/8(13%)	5/11(46%)	0.025
Epithelium				
HIF-1α	7/17 (42%)	2 (25%)	6/10 (60%)	0.323
CAIX	5 (32%)	1/7 (14%)	3/10 (30%)	0.735
VEGF	12 (67%)	5 (63%)	5/10 (50%)	0.684
Ki67	1 (6%)	0 (0%)	1/10 (10%)	0.655
p53	0(0%)	0(0%)	0(0%)	-

Due to empty blocks, repeated unsuccessful staining attempts or too little epithelium in a malignant tumor, numbers varied slightly for some stainings. Dichotomized values were used. ^a^Using the chi-square test, *P *values below 0.05 were regarded as significant. HIF-1α = hypoxia-inducible factor 1 alpha, CAIX = carbonic anhydrase IX, VEGF = vascular endothelial growth factor.

**Table 3:** Association of hypoxia-inducible factor 1 alpha (HIF-1α) with its downstream effectors, microvessel counts and proliferation markers in stroma of fibroepithelial tumors

	HIF-1α		*P *value^a^
		
	<1%	≥1%	
p53			
<10%	45	15	0.003
≥10%	1	6	
Vascular endothelial growth factor			
Weak	33	10	0.098
Strong	13	11	
Hot-spot microvessel count			
<71	25	5	0.031
≥71	20	15	
Global microvessel count			
<86	27	5	0.015
≥86	18	15	
Carbonic anhydrase IX			
Negative	45	19	0.098
Positive	0	2	
Ki67			
<10%	42	7	<0.001
≥10%	4	14	
Mitotic activity index			
<10	43	8	<0.001
≥10	3	13	

Dichotomized values were used.

^a ^Fisher's exact test, *P *values below 0.05 were regarded as significant.

**Table 4:** Prognostically significant variables in phyllodes tumors as determined by univariate analysis of disease-free survival (DFS)

Variable	Number of patients	Number of patients with disease (%)	Number of disease-free patients (%)	Mean DFS (months [95% confidence interval])	*P *value	Log-rank
p53 stroma						
<10%	24	5 (21)	19 (79)	173 (142–204)	<0.001	10.97
≥10%	6	5 (83)	1 (16)	61 (23–98)		
HIF-1α stroma						
<1%	15	3 (20)	12 (80)	180 (146–214)	0.032	4.59
≥1%	15	7 (47)	8 (53)	103 (64–142)		
Ki67 stroma						
<10%	16	4 (25)	12 (75)	176 (145–207)	0.028	4.85
≥10%	14	6 (43)	8 (57)	91 (55–127)		
Grade						
Benign	15	4 (27)	11 (73)	165 (130–200)	0.039	6.48
Borderline	5	1 (20)	4 (80)	140 (86–194)		
Malignant	10	5 (50)	5 (50)	71 (32–109)		

HIF-1α, hypoxia-inducible factor 1 alpha.
